# Back pain and erythema nodosum in a 9-year-old child

**DOI:** 10.1590/0037-8682-0318-2022

**Published:** 2022-09-30

**Authors:** Maria Francesca Gicchino, Alma Nunzia Olivieri

**Affiliations:** 1 University of the Study of Campania “Luigi Vanvitelli”, Department of Woman, Child and General and Specialized Surgery, Napoli, Italy.

A 9-year-old girl was admitted to our department with back pain and asthenia. Two weeks before the onset of back pain, the patient had experienced trauma following a fall from a swing. The patient presented with a fever (temperature up to 38°C) and lumbar pain. Initially, these symptoms were treated with non-steroidal anti-inflammatory drugs. The patient visited our department due to persistent symptoms. Clinical examination revealed tenderness in the lumbosacral column, pressure in the right sacroiliac joint, and erythema nodosum (EN) on the left leg (Figure 1). Blood examinations revealed elevated erythrocyte sedimentation rate (39 mm/h) and C-reactive protein level (3.7 mg/dL). On account of persistent back pain, we performed magnetic resonance imaging (MRI) of the spine which revealed an effusion at the level of the right sacroiliac joint (Figure 2). Due to the presence of EN, we performed the Mantoux intradermal reaction and QuantiFERON tests. The Mantoux intradermal reaction test yielded positive results at 48 hours, with an induration diameter of 25 mm. The QuantiFERON test also yielded positive results. Tuberculous sacroiliitis was diagnosed^1^ ^,^ ^2^ and treatment was initiated with ethambutol, pyrazinamide, rifampicin, and isoniazid^1^. A follow-up spinal MRI performed two months later showed no alterations (Figure 3). In children presenting with back pain, atypical causes, such as tuberculosis, should be considered to arrive at a correct diagnosis and initiate adequate treatment to prevent neurological sequelae and spinal deformity^2^ ^,^ ^3^.

**FIGURE 1:**
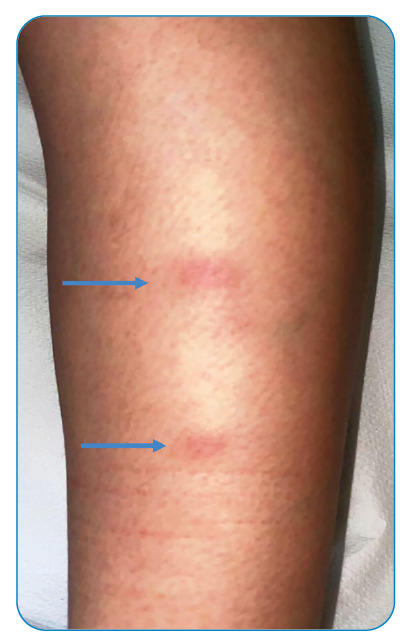
Erythema nodosum on the patient’s left leg.

**FIGURE 2:**
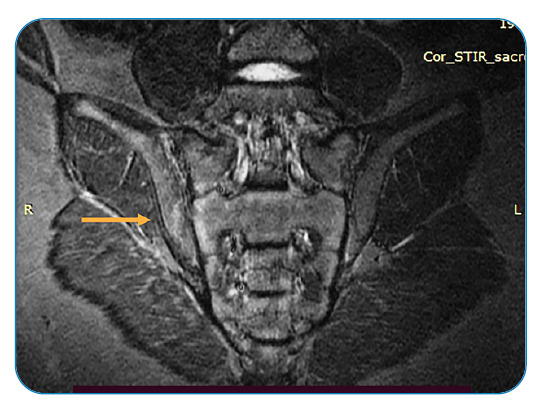
MRI showing effusion at the level of the right sacroiliac joint.

**FIGURE 3:**
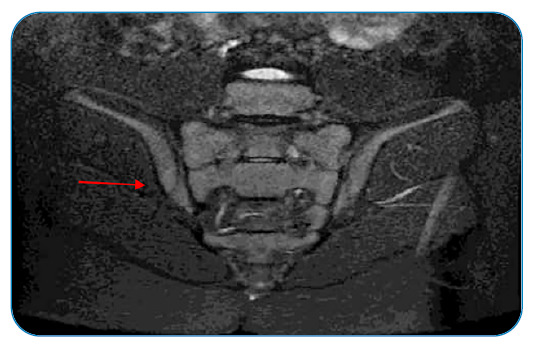
MRI showing resolution of the effusion.

## References

[1] Khanna K, Sabharwal S (2019). Spinal tuberculosis: a comprehensive review for the modern spine surgeon. Spine J.

[2] Kilborn T, Janse van Rensburg P, Candy S (2015). Pediatric and adult spinal tuberculosis: imaging and pathophysiology. Neuroimaging Clin N Am.

[3] Chatterjee S, Banta A (2018). The spectrum of tuberculosis of the spine in pediatric age group: a review. Childs Nerv Syst.

